# Tactile Sliding Behavior of R2R Mass-Produced PLLA Nanosheet towards Biomedical Device in Skin Applications

**DOI:** 10.3390/nano8040210

**Published:** 2018-03-30

**Authors:** Sheng Zhang, Yoshitomo Kai, Yuta Sunami

**Affiliations:** 1 Micro/Nano Technology Center, Tokai University, 4-1-1 Kitakaname, Hiratsuka, Kanagawa 259-1292, Japan; 2 Course of Mechanical Engineering, Tokai University, 4-1-1 Kitakaname, Hiratsuka, Kanagawa 259-1292, Japan; 6bemm017@mail.u-tokai.ac.jp; 3 Department of Mechanical Engineering, Tokai University, 4-1-1 Kitakaname, Hiratsuka, Kanagawa 259-1292, Japan

**Keywords:** tactile friction, nanosheet, sliding behavior, micro wear, tribology, PLLA, sliding

## Abstract

In this research, sliding friction was measured between the fingertip and nanosheet on a silicon substrate under two conditions: dry and wet. By using a force transducer, the tactile friction force and applied load were measured. According to the experimental results, the relationship of friction force and applied load exhibits a positive correlation under both dry and wet conditions. In addition, the nanosheets are able to reduce the friction force and coefficient of friction (COF) compared to the reference sample, especially under the wet condition. Under the assumption of a full contact condition, the estimated contact area increases with larger applied loads. Furthermore, based on the wear observation, the skin sliding performance caused slight abrasions to the surface of the nanosheet samples with a mild wear track along the sliding direction. Overall, the sliding behavior between the skin and nanosheet was investigated in terms of friction force, COF, applied load, contact area, and wear. These findings can contribute to the nanosheet-related research towards biomedical devices in skin applications.

## 1. Introduction

Ultrathin films, commonly known as nanosheets, possess a tremendously large surface-area-to-thickness ratio that has attracted increasing attentions over a great range of research fields [1]. Different from their bulk state, the two-dimensional (2D) shapes of the nanosheet state exhibit unique physical, chemical, and electronic properties, thus revealing potential applications in various areas, e.g., catalysis, sensing, biomedical fields, etc. [2,3,4]. In particular, nanosheet technology towards biomedical applications has a promising future in, e.g., drug delivery, nanoscale biodevices, wound treatment, etc. [5]. For example, nanosheets composed of versatile clinically used biomaterials possess high levels of bio-friendly adhesiveness and flexibility, which have been applied to surgical sealing operations [6]. In the case of severe tissue damage or inflammation, surgical suturing is technically difficult to conduct; however, the composition of polyester materials like poly(l-lactic acid) (PLLA) enhances nanosheets to possess sufficient adhesive strength to cover the wound site with a comparatively easier procedure. The excellent adhesiveness of PLLA nanosheets can stabilize the wound area without eliciting an inflammatory response. Okamura et al. conducted several research experiments using a PLLA nanosheet as patchwork treatment for burn wounds with no assistance of any adhesive reagent [7]. According to the results, the PLLA nanosheets attached to various wound interfaces firmly bonded together. The patchwork function also acted as an excellent barrier to prevent infection during the treatment of burns.

The fabrication technique of nanosheets is another important factor. The roll-to-toll (R2R) web handling technique has enabled the massive production of nanosheets with a defined thickness in the nanoscale range. With the ability of mass production, the cost and fabrication time of nanosheets have been greatly reduced, thus promoting the commercialization of the nanosheet.

In this paper, we investigate the sliding behavior and the tribological mechanism of R2R mass-produced PLLA nanosheets interacting with skin in vivo.

## 2. Experimental Method

### 2.1. Materials

**Figure 1:**
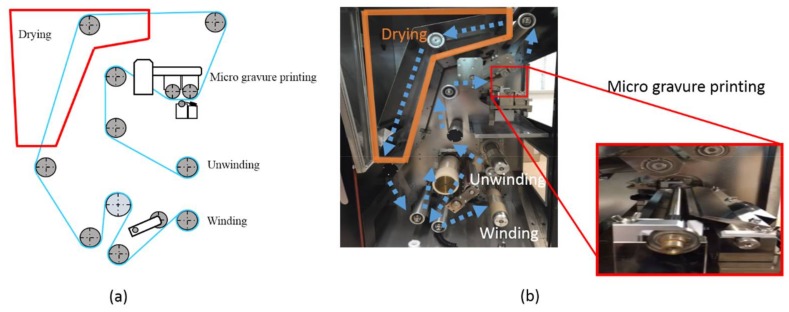
(**a**) Schematic diagram thin film coating machine; (**b**) thin film coating machine (NMC-350 YASUI SEIKI, Nagasaki, Japan).

The nanosheets used in this experiment were fabricated using a thin film coating machine (Figure 1) (NMC-350 YASUI SEIKI, Nagasaki, Japan). This thin film coating imitates the R2R production method with unwinding, winding, and drying in a furnace. In addition, micro gravure printing, which is specially employed for thin film coating, was applied in the coating process [7,8]. A nanosheet of an arbitrary film thickness can be fabricated by changing the film transporting speed, the rotational speed of the micro gravure roll, and the concentration of the solution. In addition, in this research a sacrificial film method was used to remove the nanosheets from the substrate. First, a substrate film of 100 μm was installed through a roller and tension was applied. Next, the sacrificial film, solution of Polyvinyl Alcohol (PVA), was coated on a Polyethylene Terephthalate (PET) film (T60, TORAY, Tokyo, Japan) that served as a substrate. Thereafter, it was dried at 70 °C. For the next step, a poly(l-lactic acid) (PLLA) (18402-10, Polysciences, Inc., Warrington, PA, USA) nanosheet was coated to be superimposed. By immersing the film coated with each solution in water, the sacrificial film was dissolved. Finally, without the sacrificial film, the nanosheet was peeled off. In order to obtain accurate measurements of the film thickness, both contact and non-contact measuring methods were used. In the contact method, a palpated shape measuring instrument (DektakXT, BRUKER, Morrisville, NC, USA) was used to measure the film thickness of the prepared nanosheets, resulting in a thickness of 79.4 ± 4.6 nm. In the non-contact method, a light interference microscope (BW-S500, Nikon, Tokyo, Japan) was used to measure the film thickness of the same samples, giving a thickness of 72.5 ± 10.5 nm.

### 2.2. Experimental Setup and Preparation

**Figure 2:**
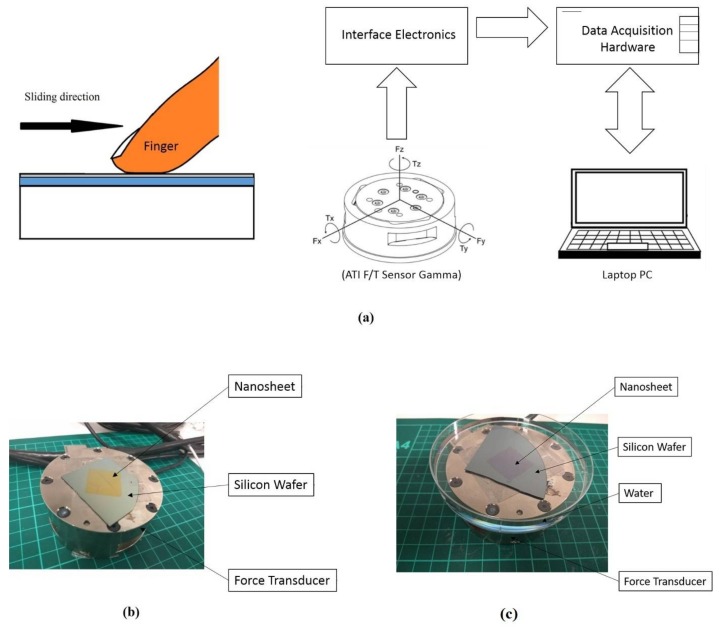
(**a**) Schematic figure of force transducer setup; (**b**) friction measurement in vivo under the dry condition; (**c**) friction measurement in vivo under the wet condition.

A force transducer (ATI F/T Sensor Gamma, ATI Industrial Automation, Apex, NC, USA) was used to measure the friction force between the nanosheet and skin in vivo (Figure 2a). The load cell device uses six degrees of freedom to measure the forces including the normal load in the *z*-axis (resolution is 25 mN) and the tangential forces in the *x* and *y* axes (resolutions are 12.5 mN) with a sampling rate of 100 Hz. Under the dry condition, the nanosheet sample with the silicon wafer substrate was fixed to the top of the force transducer using double-sided tape (Figure 2b). Under the wet condition, the sample was fixed on the container and emerged in water (Figure 2c). The index finger of the dominant hand (right hand) of a male adult (33 years old) with no known skin disease was used for all of the experiments conducted in this research. Each friction measurement was taken by sliding in the direction of the *x*-axis, and consisted of three repetitive single strokes of the finger. The stroke length was the size of the nanosheet, 20 mm. During the friction measurement, the sliding velocity was kept as constant as possible. In addition, the inclination of the finger was kept at 45° in order to maintain the nominal contact area for all experiments. Since the tactile friction measurements were conducted in vivo, it was important for the participant to conduct the sliding motion at a steady speed. Before the actual measurement, several sliding practices were performed to make sure that the participant was able to control the sliding motion at a constant velocity. All experiments were carried out in an environmentally controlled laboratory at 25 ± 1 °C. The testing finger was cleaned with soap and water and air-dried for 10 min to remove possible sweat and oil on the skin surface before each experiment.

## 3. Results and Discussion

### 3.1. Experimental Results

**Figure 3:**
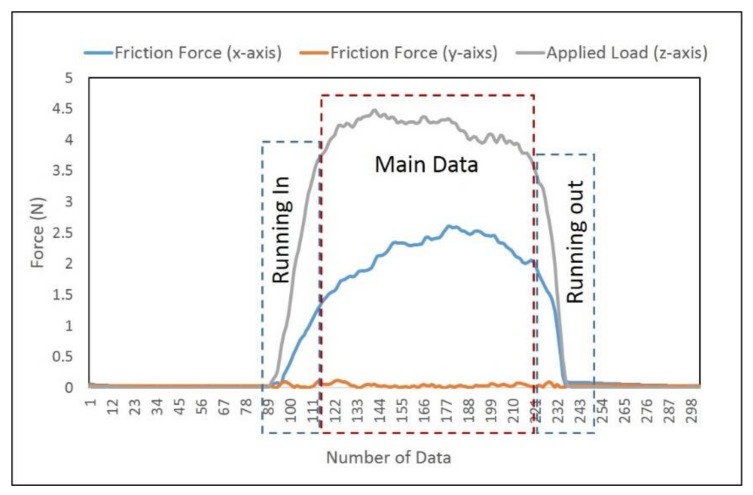
Force measurement of a single stroke.

**Figure 4:**
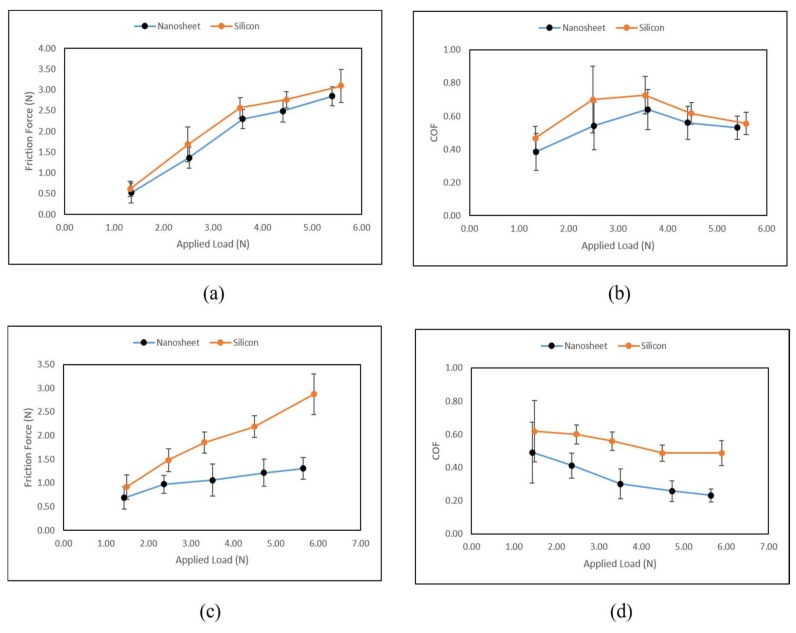
(**a**) Average friction force versus average applied load under the dry condition; (**b**) Average coefficient of friction (COF) versus applied load under the dry condition; (**c**) Average friction force versus average applied load under the wet condition; (**d**) Average COF versus applied load under the wet condition.

The raw measurement of the single stroke force on all three axes (*x*, *y*, *z*) is shown in Figure 3. Only the main data (excluding the running-in and running-out data) were used for analysis (Figure 3). The data of the running-in zone revealed a rapid increase before reaching stable data. On the contrary, the data of the running-out zone revealed a rapid decrease. In order to obtain accurate experimental results, the data of the running-in and running-out zones need to be excluded from the main data. The measured force on the *z*-axis is the applied load. Since the sliding direction was along the *x*-axis, the measured force on the *x*-axis is indicated as the friction force generated between the testing finger and sample. However, the force on the *y*-axis remains zero. For the friction measurements, the range of the resulting average normal force (applied load) on the nanosheet sample with the silicon wafer substrate was from 1.35 N to 5.41 N. The coefficient of friction (COF) was calculated based on the ratio of friction force ($F_{f}$) and applied load ($F_{N}$) as COF $=\;\frac{F_{f}}{F_{N}}$. Three repetitions were conducted by sliding the finger against the testing samples for each load range. During the in vivo tactile sliding measurements, it is difficult to control the normal load at a fixed force. However, the load range could be maintained by the participant. In this case, the normal loads are divided into five ranges which are 1–2 N, 2–3 N, 3–4 N, 4–5 N, and 5–6 N, and the average values of friction force, applied load, and COF are plotted in Figure 4. 

The experimental results are tabulated in Table 1. Under the dry condition, the positive correlation of friction force and applied load was revealed on both nanosheet samples (nanosheet with silicon wafer as substrate) and reference samples (silicon wafer only) (Figure 4a). Moreover, the nanosheets demonstrated the ability to reduce the friction force compared to the reference sample with no nanosheet. Under the wet condition, the relationship of friction force and applied load exhibited a positive correlation as well. In addition, the ability of friction deduction was more significant under the wet condition compared to the dry condition (Figure 4a,c). The same phenomena are observed in Figure 4b,d: lower values of COF on nanosheets samples were obtained compared to the reference samples under both the dry and wet conditions. Overall, the nanosheets were able to reduce the friction and COF under both conditions, being especially more effective under the wet condition. Under the dry condition, the values of COF tended to rise at first and decrease to a stable value when normal load was increased. Under the wet condition, COF constantly decreased and became stable when the normal load was increased.

**Figure 5:**
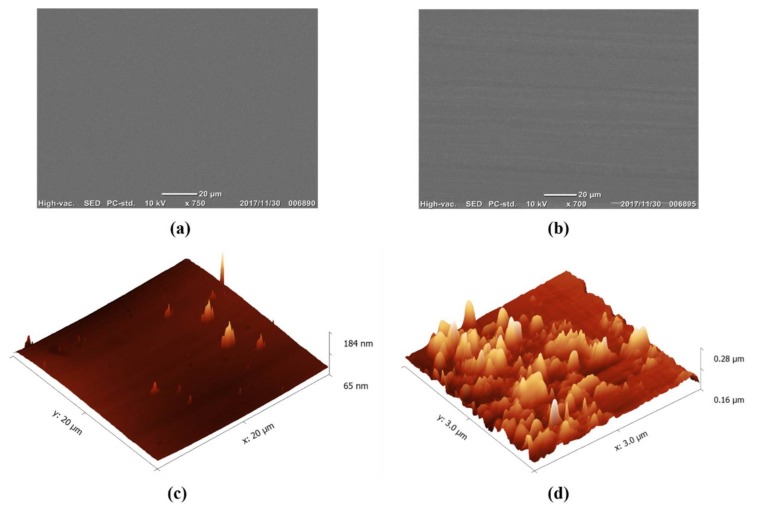
(**a**) SEM images of the nanosheet before the friction measurement and (**b**) after the friction measurement; atomic force microscopy (AFM) images of the nanosheet (**c**) before the friction measurement and (**d**) after the friction measurement.

The wear of sliding friction on the nanosheet sample was investigated. The nanosheet sample conducted with maximum normal load of 5.41 N under the dry condition was selected for the wear study. In order to investigate the sliding abrasion on the nanosheets, the scanning electron microscope (SEM) was set to 15 kV under high vacuum mode to observe the surface images of nanosheets before and after the experiments. As shown in Figure 5, the wear track caused by finger sliding was observed compared to the SEM image of the nanosheet sample before the friction measurements (Figure 5a,b).

However, the wear appears to be in nanoscale, which is beyond the scanning ability of SEM [9]. Atomic force microscopy (AFM) is considered as one of the most suitable surface measuring methods due to the extremely high possible lateral resolution of up to 0.2 nm with a vertical resolution of up to 0.01 nm [10]. In this case, to examine the change in the morphology of surface features on the nanosheet samples, an atomic force microscope (AFM) (SPM-9700, SHIMADZU, Kyoto, Japan) was used with a standard filament and probe current deposited at 3 mA. The comparison was based on the surface analysis of AFM images (before and after frictions measurements). As shown in Figure 5c,d, the surface roughness *Ra* of nanosheet changed from 2.96 nm to 9.06 nm due to the abrasion caused by finger sliding.

### 3.2. Discussion

The tactile friction (also known as skin friction) can be influenced by factors such as material properties of skin, surface texture, resulting contact area, and normal load. These factors are well-formulated as a two-term friction model, which consists of the adhesive component and deformation component of skin friction, as follows [11,12]:

(1) $$F_{f,tot}=\;\tau \cdot A_{real}+\;\frac{3}{16}\beta \frac{\delta }{a}F_{N}$$

where $F_{f,tot}$ is the total friction force; $\tau \cdot A_{real}$ is the adhesive component of friction; $\frac{3}{16}\beta \frac{\delta }{a}F_{N}$ is the deformation component of friction; τ is the interfacial shear strength; $A_{real}$ is the real contact area; β is the viscoelastic loss fraction; *δ* is the indentation of the skin; $F_{N}$ is the applied load; and *a* is the contact radius of the fingertip, which can be determined as follows:

(2) $$a=\;\sqrt[{3}]{\frac{3RF_{N}}{4E^{*}}}$$

(3) $$\frac{1}{E^{*}}=\;\frac{1-\;\nu_{finger}^{2}}{E_{finger}}+\;\frac{1-\;\nu_{surface}^{2}}{E_{surface}}$$

where *R* is the radius of the fingertip; E* is the effective Young’s modulus; $\;E_{finger}$ and $E_{surface}$ are the Young’s moduli of the finger and counter-surface; and $\nu_{finger\;}$ and $\nu_{surface}$ are the Poisson’s ratios of the finger and counter-surface, respectively.

**Figure 6:**
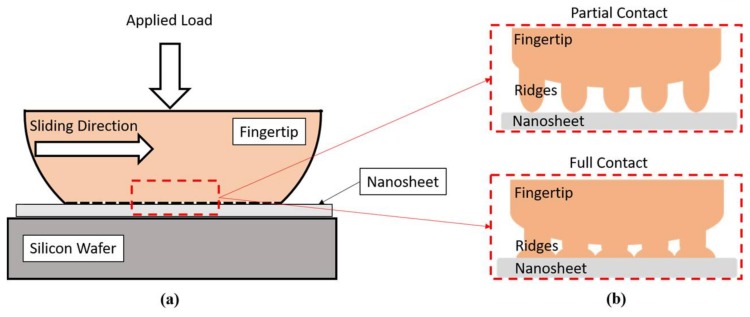
(**a**) Schematic figure of contact mechanism; (**b**) partial contact and full contact conditions.

**Figure 7:**
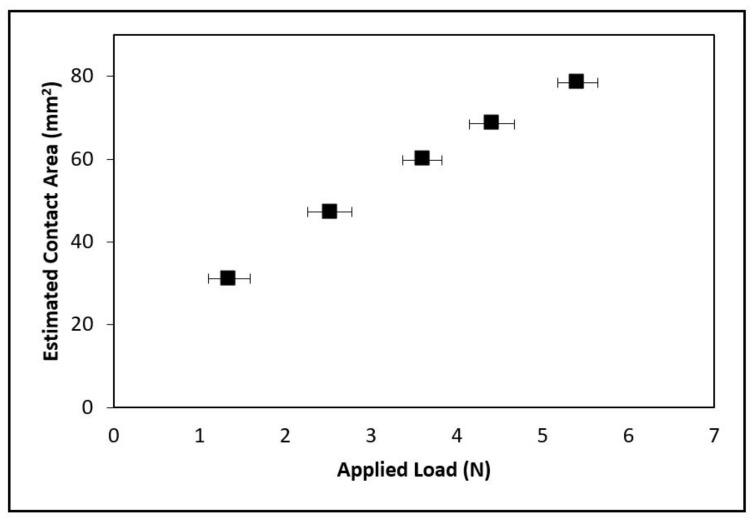
Estimated contact area versus applied load.

The surface roughness of the nanosheet (nanoscale) is much smaller compared to the finger’s ridges (microscale). Therefore, the contact radius depends mainly on the radius of the testing fingertip (Figure 6). According to Equations (2) and (3), the contact radius and the contact area can be predicted in conjunction with the parameters obtained from the literature (Table 2). As shown in Figure 6, the finger ridges (fingerprint) is much rougher than the nanosheet sample. In other words, the deformation component of the friction greatly relies on the indentation of the fingerprint. According to the research conducted by Warman and Ennos, the fingerprint is able to reduce the contact area by 33% compared with flat skin [13]. To best describe this wavy-type deformation, the Westergaard contact model can be applied in this case [14,15,16]:

(4) $$\frac{\left(\lambda -w\right)}{\lambda }=\;\frac{2}{\pi }\;\sin^{-1}{\left(\frac{\bar{p}}{p^{*}}\right)}^{1/2}$$

where  λ is the spacing between the ridges of the fingerprint; w is the width of a ridge; $\bar{p}$ is the actual surface pressure; $p^{*}$ is the pressure needed for the finger under the full contact condition. The contact area is influenced by the contact condition, which is determined by the pressure ratio ($\frac{\bar{p}}{p^{*}}$). When the pressure ratio ($\frac{\bar{p}}{p^{*}}$) is less than 1, the contact area of ridges is under the partial contact condition. When the pressure ratio ($\frac{\bar{p}}{p^{*}}$) is greater than or equal to 1, the contact area is under the full contact condition (Figure 6b). More detailed investigation towards the concerns of surface texture and surface roughness can be found in a previous research work [15]. In this research, the full contact condition was assumed, and the relationship of the estimated contact area and applied loads are plotted in Figure 7. The estimated contact area between the fingertip and nanosheet increased when the applied load was increased. 

## 4. Conclusions

In this research, sliding friction was measured between the fingertip and nanosheet on a silicon substrate under two conditions: dry and wet. According to the experimental results, the relationship between the friction force and applied load exhibits a positive correlation under both the dry and wet conditions. In addition, the nanosheets are able to reduce the friction force and COF compared to the reference sample, especially under the wet condition. Moreover, with the assumption of a full contact condition, the estimated contact area increases with larger applied loads. Based on the wear observation, the skin sliding performance was not able to cause severe damage to the surface of nanosheet samples; however, the mild wear track was observed along with the sliding direction. Overall, the sliding behavior between skin and nanosheet was investigated in terms of friction force, COF, applied load, contact area, and wear. The findings in this paper can contribute to nanosheet-related research towards biomedical devices developed for skin applications.

## Figures and Tables

**Table 1:** Experimental data of friction measurements under the dry and wet conditions.

Contrasting Conditions	Samples	Friction (N)	Normal Load (N)	COF
Dry condition	Nanosheet	0.52 ± 0.12	1.35 ± 0.24	0.39 ± 0.11
	Nanosheet	1.36 ± 0.38	2.52 ± 0.26	0.54 ± 0.14
	Nanosheet	2.30 ± 0.38	3.60 ± 0.23	0.64 ± 0.12
	Nanosheet	2.49 ± 0.41	4.41 ± 0.26	0.56 ± 0.10
	Nanosheet	2.85 ± 0.35	5.41 ± 0.23	0.53 ± 0.07
	Silicon wafer	0.62 ± 0.09	1.33 ± 0.18	0.47 ± 0.07
	Silicon wafer	1.68 ± 0.39	2.49 ± 0.42	0.7 ± 0.2
	Silicon wafer	2.57 ± 0.40	3.55 ± 0.25	0.73 ± 0.11
	Silicon wafer	2.76 ± 0.35	4.48 ± 0.20	0.62 ± 0.07
	Silicon wafer	3.10 ± 0.35	5.59 ± 0.40	0.56 ± 0.07
Wet Condition	Nanosheet	0.68 ± 0.24	1.43 ± 0.20	0.49 ± 0.18
	Nanosheet	0.97 ± 0.19	2.36 ± 0.18	0.41 ± 0.08
	Nanosheet	1.06 ± 0.34	3.51 ± 0.24	0.30 ± 0.09
	Nanosheet	1.21 ± 0.28	4.72 ± 0.35	0.26 ± 0.06
	Nanosheet	1.31 ± 0.23	5.65 ± 0.41	0.23 ± 0.04
	Silicon wafer	0.92 ± 0.29	1.49 ± 0.26	0.62 ± 0.19
	Silicon wafer	1.48 ± 0.18	2.47 ± 0.24	0.60 ± 0.06
	Silicon wafer	1.85 ± 0.24	3.32 ± 0.22	0.56 ± 0.05
	Silicon wafer	2.19 ± 0.25	4.50 ± 0.23	0.49 ± 0.05
	Silicon wafer	2.88 ± 0.53	5.90 ± 0.43	0.49 ± 0.08

**Table 2:** Parameters from the literature are used in the analytical model to estimate the contact area between the finger and counter-surface [17,18,19].

Properties	Values
*E_finger_*	0.2 MPa
*E_surface_*	150 GPa
*v_finger_*	0.48
*v_surface_*	0.28
